# Vitamin A Deficiency Impairs Adaptive B and T Cell Responses to a Prototype Monovalent Attenuated Human Rotavirus Vaccine and Virulent Human Rotavirus Challenge in a Gnotobiotic Piglet Model

**DOI:** 10.1371/journal.pone.0082966

**Published:** 2013-12-02

**Authors:** Kuldeep S. Chattha, Sukumar Kandasamy, Anastasia N. Vlasova, Linda J. Saif

**Affiliations:** The Food Animal Health Research Program, Department of Veterinary Preventive Medicine, Ohio Agricultural Research and Development Center, The Ohio State University, Wooster, Ohio, United States of America; Nanyang Technological University, Singapore

## Abstract

Rotaviruses (RV) are a major cause of gastroenteritis in children. Widespread vitamin A deficiency is associated with reduced efficacy of vaccines and higher incidence of diarrheal infections in children in developing countries. We established a vitamin A deficient (VAD) gnotobiotic piglet model that mimics subclinical vitamin A deficiency in children to study its effects on an oral human rotavirus (HRV) vaccine and virulent HRV challenge. Piglets derived from VAD and vitamin A sufficient (VAS) sows were orally vaccinated with attenuated HRV or mock, with/without supplemental vitamin A and challenged with virulent HRV. Unvaccinated VAD control piglets had significantly lower hepatic vitamin A, higher severity and duration of diarrhea and HRV fecal shedding post-challenge as compared to VAS control pigs. Reduced protection coincided with significantly higher innate (IFNα) cytokine and CD8 T cell frequencies in the blood and intestinal tissues, higher pro-inflammatory (IL12) and 2-3 fold lower anti-inflammatory (IL10) cytokines, in VAD compared to VAS control pigs. Vaccinated VAD pigs had higher diarrhea severity scores compared to vaccinated VAS pigs, which coincided with lower serum IgA HRV antibody titers and significantly lower intestinal IgA antibody secreting cells post-challenge in the former groups suggesting lower anamnestic responses. A trend for higher serum HRV IgG antibodies was observed in VAD vs VAS vaccinated groups post-challenge. The vaccinated VAD (non-vitamin A supplemented) pigs had significantly higher serum IL12 (PID2) and IFNγ (PID6) compared to vaccinated VAS groups suggesting higher Th1 responses in VAD conditions. Furthermore, regulatory T-cell responses were compromised in VAD pigs. Supplemental vitamin A in VAD pigs did not fully restore the dysregulated immune responses to AttHRV vaccine or moderate virulent HRV diarrhea. Our findings suggest that that VAD in children in developing countries may partially contribute to more severe rotavirus infection and lower HRV vaccine efficacy.

## Introduction

Rotavirus (RV) is a leading cause of severe viral gastroenteritis in infants and young children. Annually RV diarrhea is responsible for more than 450,000 deaths worldwide among children less than 5 years of age. The majority of deaths occur in developing countries of Asia and Africa [1]. In the United States, RV infection accounts for more than $1 billion in health related costs. Two attenuated oral RV vaccines (RotaTeq [Merck] and Rotarix [Glaxo Smithkline Biologicals]) are available, which have been recommended by the World Health Organization (WHO) to be included in the national immunization programs of all countries worldwide [2,3]. These new second generation RV vaccines have improved the efficacy (~50%) against severe RV diarrhea compared to first generation vaccines (~20%) in low income countries [2]. However, their efficacy in low income, where RV infection is prevalent, is significantly lower than in middle and high income countries [2,4]. The precise reasons for these differences in vaccine efficacy between developed and developing countries are unknown.

In low income countries, widespread malnutrition and micronutrient deficiencies including vitamin A deficiency, and higher prevalence of RV infections may result in higher RV associated morbidity and mortality, and also in lower vaccine efficacy. Almost 33% of all preschool children globally are vitamin A deficient (VAD) with the majority of these cases occurring in Africa and Southeast Asia (44.4% and 49.9% of all preschool children, respectively) [5]. Moreover, only 55% of the target population receives supplemental vitamin A [6]. To improve vitamin A status and reduce disease severity, WHO has recommended supplementation of vitamin A in VAD endemic areas as part of an expanded program of immunization [5]. WHO recommends 100,000 IU (30 mg) of vitamin A for children 6-11 months of age along with oral polio, measles and DPT vaccines [5]. Supplementation of vitamin A in children has decreased mortality and morbidity associated with diarrhea irrespective of infectious agent [7]. *In vivo* (clinical and animal models) and *in vitro* studies have shown that vitamin A is a key regulator of gut immunity and thus may affect immune responses to oral vaccines and mucosal infections [8,9].

Retinoic acid (RA), an immunologically active metabolite of vitamin A, has been shown to mediate mucosal homing (α4β7 and CCR9) of B and T cells, generation of T regulatory (Treg) cells and enhance immunoglobulin (Ig) A antibody and antibody secreting cell (ASC) responses[10-12]. These immunomodulatory effects of RA are primarily mediated through gut dendritic cells (DCs), which play a central role in generation of appropriate gut immune responses [8,11]. These studies have shown a general role of vitamin A in regulating gut mucosal immune responses. However, only a few studies have investigated systemic and mucosal B and T cell responses to specific pathogens or vaccines in experimental VAD conditions, for example Newcastle disease virus [13], *Escherichia coli* [14], recombinant human immunodeficiency virus vaccine [15], cholera vaccine [16], influenza virus [17], Sendai virus [18,19], tetanus toxoid [20]. Vitamin A also exhibits non-immune mediated effects on mucosal surfaces and is required for maintaining the integrity of epithelial surfaces [9,21] and thus may play an important role in recovery from RV diarrhea. Previous studies of RV infected adult VAD mouse models (generated by dietary vitamin A restriction) showed higher intestinal epithelial cell damage and lower serum HRV antibody responses, but no significant differences in the numbers of B and T lymphocytes in spleen compared to pair-fed mice [22,23]. However, RV causes severe infection in neonates and VAD is more prevalent in children as compared to adults. Therefore, in this study we measured the impact of VAD and supplemental vitamin A on RV vaccine and infection in a neonatal gnotobiotic pig model of human rotavirus (HRV) diarrhea. 

Gnotobiotic pigs show clinical signs of diarrhea and virus shedding post-HRV challenge similar to infants and their physiology (vitamin A metabolism) and immune responses mimic those of humans [24-27]. Thus, neonatal VAD gnotobiotic pigs are a relevant animal model to investigate interaction of attenuated HRV (AttHRV) vaccine and virulent HRV infection and vitamin A supplementation. Moreover, being outbred, pigs exhibit heterogeneity in immune responses similar to humans. Studies of supplementation of vitamin A in children have shown enhancement of antibody responses to oral polio virus vaccine and systemic measles and DPT vaccines [28,29], when vaccines were administered concurrently with vitamin A. In contrast, other studies in children have shown no change or reduced antibody responses in vitamin A supplemented children compared to non-supplemented children [7,30,31]. Previous clinical studies did not investigate the effect of VAD or supplementation on generation of intestinal B and T cell responses. Furthermore, the clinical studies were primarily based on antibody seroconversion as the only parameter examined. Thus the impact of vitamin A deficiency or vitamin A supplementation on comprehensive immune responses has not been well defined. The recent introduction of rotavirus vaccine in national immunization programs and the administration of oral vitamin A in various countries make it timely to examine the adjuvant effect of vitamin A on mucosal and systemic immune responses to RV vaccines. To examine the synergism between oral vitamin A supplementation (as recommended by WHO) and AttHRV vaccines and their effects on virulent HRV challenge, we established a VAD pig model, mimicking infants and children with subclinical vitamin A deficiency in developing countries.

## Materials and Methods

### Virus inocula

The cell culture-adapted AttHRV G1P[8] Wa and virulent HRV G1P[8] Wa strain were used for vaccination and challenge, respectively, as described previously [32]. The AttHRV was used as vaccine at a dose of 5 X 10^7^ fluorescent foci forming units (FFU) and virulent HRV was used for challenge at a dose of 1 X 10^5^ FFU (ID_50_ ~ 1FFU). 

### Ethics Statement

This study was conducted in compliance with the recommendations by Public Health Service Policy, United States Department of Agriculture Regulations, the National Research Council's Guide for the Care and Use of Laboratory Animals, and the Federation of Animal Science Societies' Guide for the Care and Use of Agricultural Animals in Agricultural Research and Teaching, and all relevant institutional, state, and federal regulations and policies regarding animal care and use at The Ohio State University. The experimental procedures were approved The Ohio State University Institutional Animal Care and Use Committee (IACUC protocol number: 2010A0088). The minimum number of pigs per treatment group that would permit detection of statistically significant differences was verified by a statistician before the experiments.

### VAD pigs and experimental design

The VAD and vitamin A sufficient (VAS) pigs were derived from VAD and VAS sows, respectively, as previously described [33]. Briefly, piglets from VAS (n=43) and VAD (n=41) sows were assigned to 1 of 4 treatment groups: Group 1- 3X AttHRV Wa vaccine (**Vac** [VAD n=12, VAS n=13]), Group 2- AttHRV Wa + Vitamin A 100,000 IU oral (**Vac+VitA** [VAD n=11, VAS n=13]), Group 3 - Placebo control (PBS, no supplemental Vitamin A) (**Ctrl** [VAD n=9, VAS n=9]) and Group 4 - Control (PBS) + Vitamin A 100,000 IU oral (**Ctrl+VitA** [VAD n=9, VAS n=8]). Both VAD and VAS pigs were fed ultra-high temperature processed commercial cow milk (Parmalat) throughout the trial. The experimental design has been described previously [33]. Briefly, vaccinated groups were orally inoculated with AttHRV Wa at post-inoculation day (PID) 0 (6 day old), PID10 and PID20. Supplemental retinyl palmitate (vitamin A) at 100,000 IU dose was given orally with each vaccine dose. Subsets of piglets were humanely euthanized on PID26 (pre-challenge) and the remaining piglets were challenged and euthanized on PID36/post-challenge day 10 (PCD10). Intestinal tissues (ileum and duodenum), spleen and blood were collected aseptically at euthanasia as previously described [34,35].

### Detection of fecal HRV shedding and assessment of clinical signs

Diarrhea scores were recorded daily post-challenge as previously described. Rectal swabs were collected daily post-virulent HRV challenge to detect fecal HRV shedding by cell culture immunofluorescence as previously described [32,33].

### Serum and liver vitamin A levels

Serum and liver samples were collected pre-and post-challenge and were submitted to the Diagnostic Center for Population and Animal Health, Michigan State University, Lansing, MI to assess vitamin A concentrations by high-performance liquid chromatography (HPLC) [33].

### HRV specific and total antibody and HRV specific ASC responses

The HRV-specific antibody titers in serum and intestinal contents were detected as previously described [34,36]. Briefly, 96-well plates (Nunc-Maxisorp) were coated overnight at 4 °C with guinea pig hyperimmune serum against group A RV. Subsequently plates were blocked with 2% milk in phosphate buffered saline containing 0.1% tween-20 for 1 h at 37 °C. Semi-purified Wa HRV or mock-infected MA-104 cell supernatants were added to the alternate columns of plates. Serial 4-fold dilutions of each test sample were added to the antigen-coated or mock antigen-coated wells and incubated for 2 h at 37 °C. For HRV IgA and IgG antibody detection, goat anti-pig IgA HRP (1:3000, AA140P; Serotec) and biotinylated goat anti-pig IgG (gamma chain) (1:20000. 16-14-02, KPL) were used, respectively. Mouse anti-pig IgG1 (MCA635, Serotec) and IgG2 (MCA636, Serotec) monoclonal antibodies followed by rat anti-mouse IgG1 HRP (559626, BD Pharmingen) were were used to detect HRV specific IgG1 and IgG2 antibody titers, respectively. The ELISA antibody titre (geometric mean titers [GMTs]) was expressed as the reciprocal of the highest dilution that had a corrected OD values (sample OD in the virus-coated well minus sample OD in the mock antigen-coated well) greater than the cut-off value (mean raw OD values of the positive capture of the negative samples + 2 X standard diaviations of the OD values of the positive capture of the negative samples). The total IgA and IgG titers were detected in serum and intestinal contents as previously described [27,34].

The HRV-specific IgA and IgG ASC in blood, spleen and intestinal tissues were investigated and enumerated as described previously [34,35]. Briefly, AttHRV Wa infected MA-104 cells, fixed with 80% acetone in 96 well flat-bottom plates (Nunc-Immuno) were used to measure HRV specific IgA and IgG ASC. The mononuclear cells (MNCs) from intestinal tissues were added to duplicate wells (5 × 10^5^, 5 × 10^4^, or 5 × 10^3^ cells/well). Plates were incubated at 37 °C for 12 h in presence of 5% CO_2_. Subsequently biotinylated mouse monoclonal antibody (ascites fluid) to pig IgG (hybridoma 3H7) and goat anti-pig IgA HRP were added for detecting HRV IgA and IgG ASC. The HRP-conjugated streptavidin was added to the wells that received pig IgG antibody. The plates were washed and spots were developed with a tetramethylbenzidine peroxidase substrate system (KPL) containing membrane developer.

### Flow cytometry

The MNCs were isolated from blood, spleen, duodenum and ileum as previously described [34,35]. The frequency of B cells among MNCs were determined as previously described [34]. The frequencies of CD3^+^CD4^+^ and CD3^+^CD8^+^ T cells among MNCs and CD4^+^CD25^+^Foxp3^+^ T cells among CD4 T cells were measured as previously described [37].

### Detection of cytokines in serum by ELISA

Blood was collected at multiple time-points pre- and post-challenge. Serum was separated by centrifugation (1,850g, 15 min) and stored at -20 °C until tested. The Th1 (IL12 [R&D Systems], IFNγ [Invitrogen]), Th2 (IL4 [Invitrogen]), Th3 and Treg (TGFβ [Invitrogen], IL10 [Invitrogen]), Th17 (IL17 [Kingfisher Biotechnologies]) and innate cytokines and chemokines (IFNα [PBL Interferon Source], IL8 [Bethyl Laboratories]) were measured to determine type of immune response. Cytokine-specific ELISAs were performed and concentrations determined as previously described [34,37].

### Statistical Analysis

Statistical analysis was done using SAS version 9.3 (SAS Institute, Cary, USA). The mean duration of diarrhea and fecal HRV shedding post-challenge, log transformed total Ig and HRV-specific antibodies were compared using one-way analysis of variance (ANOVA-general linear model), followed by Duncans multiple range test. The area under the curve (AUC) for diarrhea severity and shedding were calculated from diarrhea score-and shedding time graph of individual animals [37]. Frequencies of cells determined by flow cytometry, cytokine concentrations and AUC for diarrhea severity between and within groups were compared using Kruskal-Wallis rank sum test. Differences were considered significant at p≤0.05.

## Results

### VAD pigs had lower hepatic vitamin A levels

In this study, VAD and VAS status was confirmed by measuring hepatic vitamin A levels in the deficient and sufficient pigs for all treatment groups, which were reported previously [33]. Briefly, VAD groups had significantly lower hepatic vitamin A levels compared to counterpart VAS groups [33]. Moreover, supplementation of vitamin A in deficient and sufficient pigs significantly increased hepatic vitamin A levels at both pre and post-challenge time-points [33]. 

### VAD pigs had higher fecal HRV shedding and diarrhea severity post-challenge

**Figure 1:**
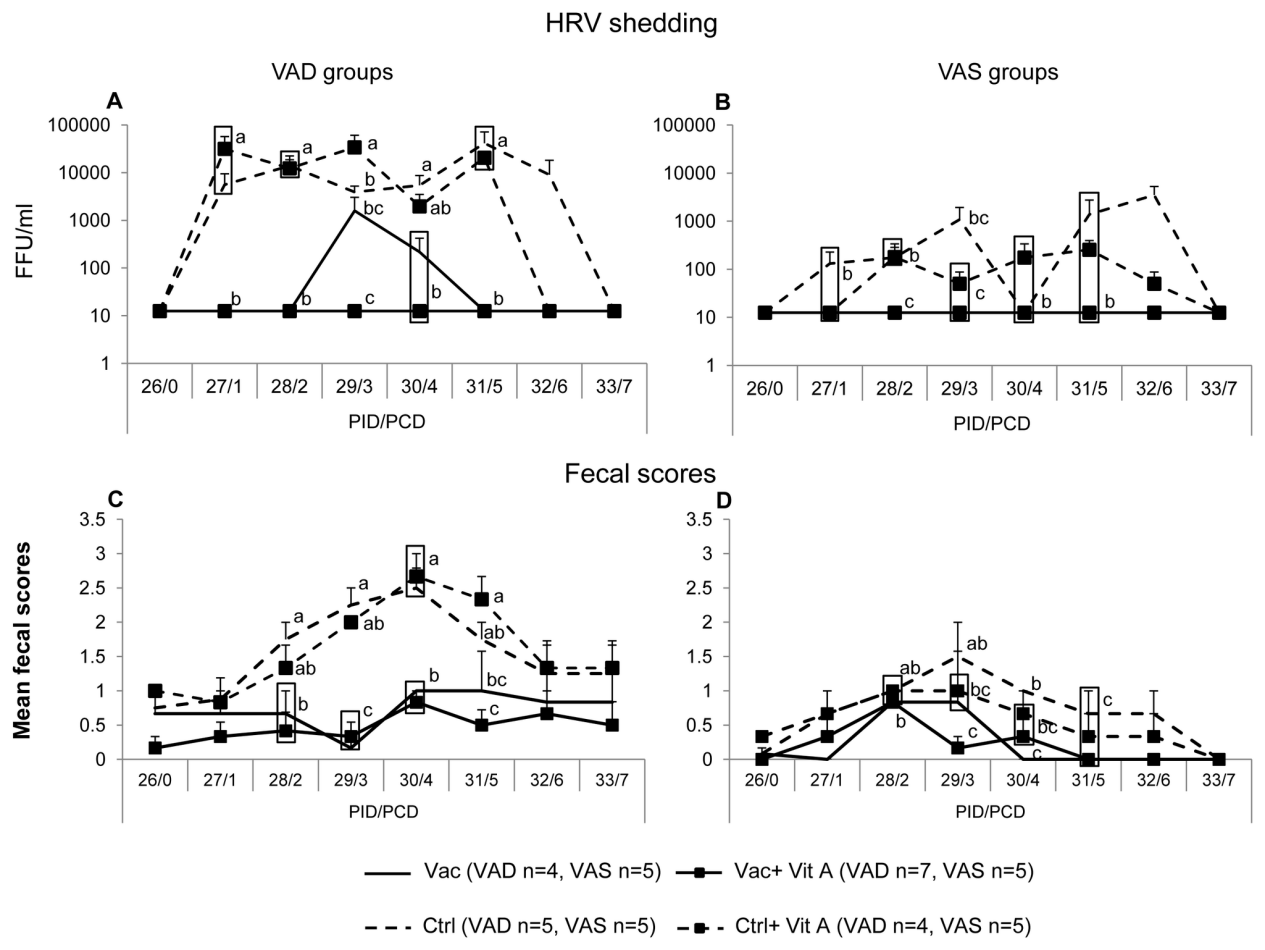
Vitamin A deficient control groups had higher fecal diarrhea scores and fecal HRV shedding post-virulent HRV challenge. Mean HRV fecal shedding (A,B) and fecal scores (C,D) post-virulent HRV challenge in vaccinated and control vitamin A deficient (VAD) and sufficient (VAS) groups. Different lower-case letters indicate statistically significant difference (one-way ANOVA followed by Duncan’s multiple range test, *p* ≤ 0.05) among the treatment groups belonging to VAD and VAS pigs at the same time-point. Bars at each time-point indicate standard error of the means. Vac = 3X AttHRV vaccinated only, Vac+VitA = 3X AttHRV vaccinated + 100,000 IU of vitamin A, Ctrl = non-vaccinated and non-vitamin A supplemented, Ctrl+VitA = 3X 100,000 IU of vitamin A only.

The VAD control pigs irrespective of vitamin A supplementation had significantly higher fecal HRV shedding titers at PCD1, PCD2 and PCD5 and higher at PCD3 and PCD4 compared to the VAS control ± vitamin A supplemented groups (Figure 1A). The AUC for diarrhea severity and shedding was significantly lower in the VAD control groups compared to the VAS control groups suggesting that vitamin A deficiency may result in more severe HRV infection and diarrhea (Table 1). Neither of the VAD control groups were protected against HRV shedding post-challenge; however, supplementation of vitamin A in the control VAD group reduced fecal HRV shedding by 1 day (Figure 1A). Similarly, supplementation of vitamin A in the control VAS group resulted in lower (but not significantly) mean fecal HRV shedding at PCD 1,3,5 and 6 suggesting some beneficial effects of supplemental vitamin A (Figure 1B). All vaccinated groups, irrespective of vitamin A status and/or vitamin A supplementation, were completely protected from fecal HRV shedding post-challenge, except the Vac VAD group, which shed HRV at PCD3 and PCD4, suggesting that vitamin A deficiency may affect vaccine efficacy against virulent HRV challenge (Figure 1A, 1B).

**Table 1:** Diarrhea severity and HRV shedding post-challenge in VAD and VAS groups^*^.

			HRV shedding		Diarrhea**^†^**	
Vitamin A status	Treatment groups	Number	Mean duration (in days)**^‡^**	AUC for shedding**^δ^**	Mean duration (in days)**^‡^**	AUC for diarrhea severity**^δ^**
VAD	Vac	4	1.3^a^	1875±1664^a^	0^a^	5.1±1.3^ad^
	Vac+VitA	7	0^a^	92±3^a^	0^a^	3.4±1.0^abd^
	Ctrl	5	5^b^	76643±37330^b^	3.5^b^	11.4±1.4^c^
	Ctrl+VitA	4	4.3^b^	89713±61900^b^	4.5^b^	11.7±0.9^c^
VAS	Vac	5	0^a^	92±4^a^	0^a^	1.7±0.4^b^
	Vac+VitA	5	0^a^	92±4^a^	0^a^	1.7±0.3^b^
	Ctrl	5	2.67^ab^	6271±2825^c^	0.33^c^	5.5±1.0^d^
	Ctrl+VitA	5	3.67^ab^	738±346^c^	0.33^c^	4.2±0.2^d^

^*^ Details for protection rates against shedding and diarrhea have been reported previously [33].

^†^ Pigs with fecal score of >1 were considered diarrheic. Fecal consistency was scored as follows: 0, normal; 1, pasty; 2, semi liquid and 3, liquid.

^‡^ Means with different superscript lower-case letters in the same column differ significantly (determined by one-way ANOVA followed by Duncan’s multiple range test).

^δ^ AUC: Area under the curve for shedding and diarrhea severity. Means with different superscript lower-case letters in the same column differ significantly (determined by Kruskal-Wallis rank sum test)

The control VAD groups, irrespective of vitamin A supplementation, had higher mean diarrhea scores (mean fecal score >1.5) from PCD2 through PCD5, and had significantly higher diarrhea severity scores (as indicated by AUC) compared to the control VAS groups ± supplemental vitamin A (mean fecal score <1.5) (Figure 1C, 1D, Table 1), which coincided with the higher fecal HRV shedding in VAD control groups. None of the vaccinated groups, irrespective of vitamin A status and/or vitamin A supplementation, differed significantly for mean fecal scores (Figure 1C, 1D), but diarrhea severity scores (AUC for diarrhea severity) were significantly higher in the Vac VAD group compared to the vaccinated VAS groups (Table 1).

### HRV IgA responses in vaccinated groups depended on the vitamin A status

#### 1. Pre-challenge

**Figure 2:**
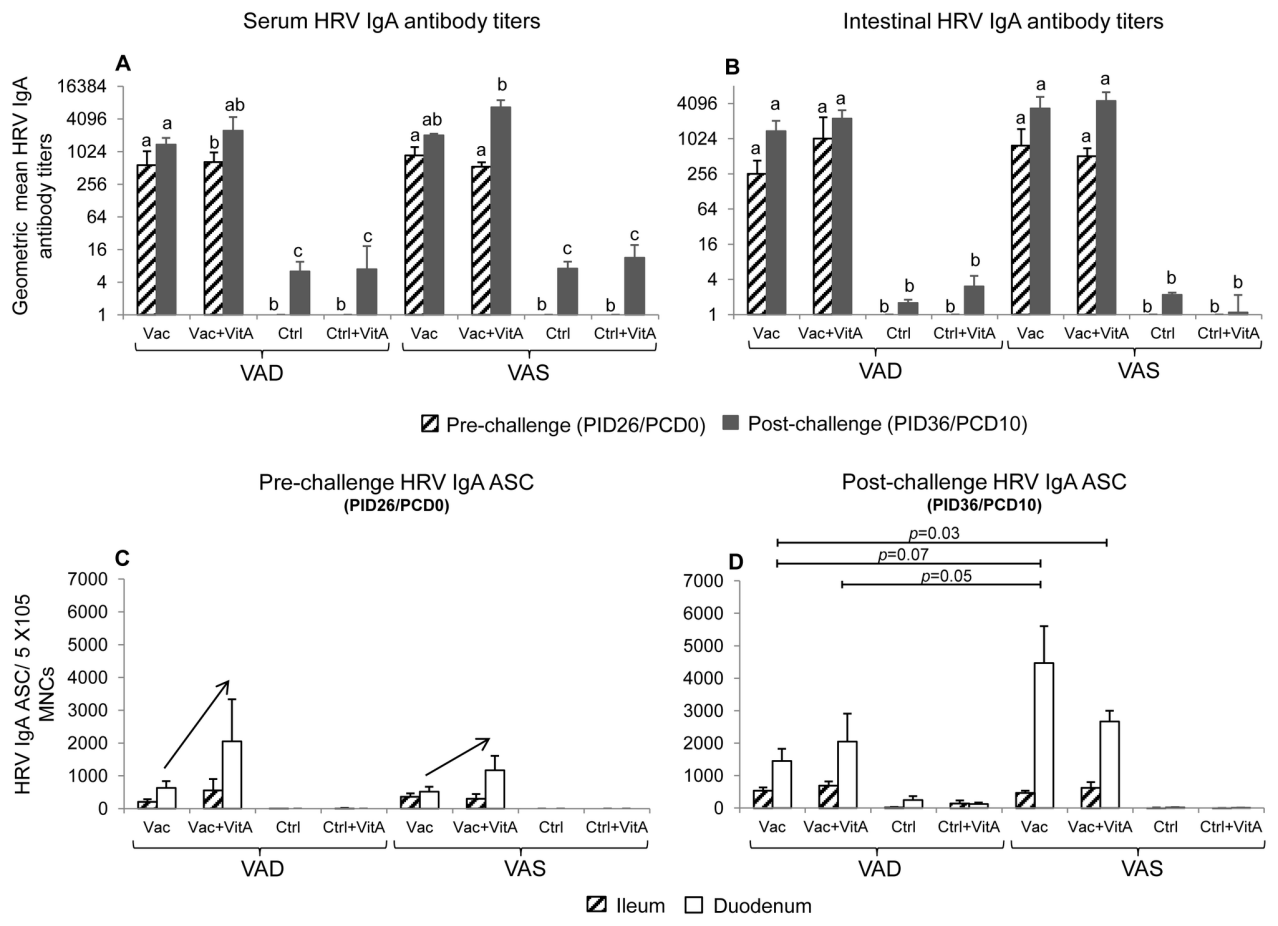
Vitamin A status affected HRV IgA antibody responses post-virulent HRV challenge. Geometric mean serum (A) and intestinal (B) HRV IgA antibody titers and mean intestinal HRV IgA antibody secreting cells (C,D) in vitamin A deficient (VAD) and sufficient (VAS) gnotobiotic pigs vaccinated with AttHRV vaccine or placebo with or without vitamin A supplementation at pre- (PID26/PCD0) and post (PID36/PCD10)-HRV challenge time-points. Data shown as mean values ± standard error of the mean. Bars with different lower-case letters for HRV IgA antibody titers (A,B) at each time-point (pre- or post-challenge) differ significantly between groups (Duncans multiple range t test on log_10_ transformed data, *p* ≤ 0.05). Significant differences between groups for HRV IgA ASC are indicated by capped lines as determined by non-parametric Kruskal-wallis rank sum test (*p* ≤ 0.05). The arrow (C) indicates increased duodenal IgA ASC in Vac+VitA group compared to Vac group for both VAD and VAS pigs at PID26/PCD0 (pre-challenge). Vac = 3X AttHRV vaccinated only, Vac+VitA = 3X AttHRV vaccinated + 100,000 IU of vitamin A, Ctrl = non-vaccinated and non-vitamin A supplemented, Ctrl+VitA = 3X 100,000 IU of vitamin A only. Pre-challenge: n=8-13 (serum IgA antibodies), 3-7 (intestinal IgA antibodies and ASC), Post-challenge: n=4-7 (serum and intestinal IgA antibodies and ASC).

At PID26/PCD0, no differences in mean serum and intestinal IgA HRV antibody titers were observed between the AttHRV vaccinated VAD and VAS groups, irrespective of vitamin A supplementation, suggesting that vitamin A deficiency may not affect induction and generation of primary antibody responses (Figure 2A, 2B). Similarly, no significant differences were observed in intestinal IgA HRV ASC between the different vaccinated groups at PID26/PCD0 (Figure 2C). However, supplementation of vitamin A in both VAD and VAS vaccinated groups increased mean duodenal IgA ASC by 2-3 fold compared to counterpart non-supplemented VAD and VAS vaccinated groups (Figure 2C).

#### 2. Post-challenge

At PID36/PCD10, vaccinated VAD groups had lower overall serum and intestinal IgA HRV antibody titers and IgA HRV ASC compared to vaccinated VAS groups (Figure 2). The Vac VAD group had significantly lower serum (Figure 2A) and a trend for lower intestinal HRV IgA antibody titers (Figure 2B), and significantly lower duodenal IgA HRV ASC (Figure 2D) compared to the Vac+VitA VAS group. These findings indicate an impact of vitamin A in groups that had maximum differences in hepatic vitamin A levels, suggesting that vitamin A levels mainly affects anamnestic antibody responses post-virulent HRV challenge. The Vac+VitA VAD and Vac VAS group also had a trend for lower serum IgA HRV antibody titers compared to Vac+VitA VAS group at PID36/PCD10 (Figure 2A). However, no significant differences were observed post-challenge between vaccinated VAS groups for duodenal and ileal IgA ASC irrespective of vitamin A supplementation (Figure 2C, 2D). Post-challenge, vaccinated VAD groups, irrespective of vitamin A supplementation, had lower duodenal IgA ASC compared to the Vac VAS group (Figure 2D). The effect of VAD on duodenal, but not on ileal IgA ASC (no difference in VAD vs VAS vaccinated groups) indicate a different impact of vitamin A in ileum, containing the peyers patches (inductive and effector site) and duodenum (effector site) (Figure 2C, 2D) suggesting that vitamin A may affect homing of ASC to duodenum. No significant differences for total serum and intestinal IgA were observed between VAD and VAS groups irrespective of vaccination, virulent HRV challenge and vitamin A supplementation (data not shown), suggesting that vitamin A deficiency affects mainly the HRV (antigen)- specific IgA antibody responses.

### Vaccinated VAD groups had lower HRV IgG antibody titers and IgG1:IgG2 antibody ratios

**Figure 3:**
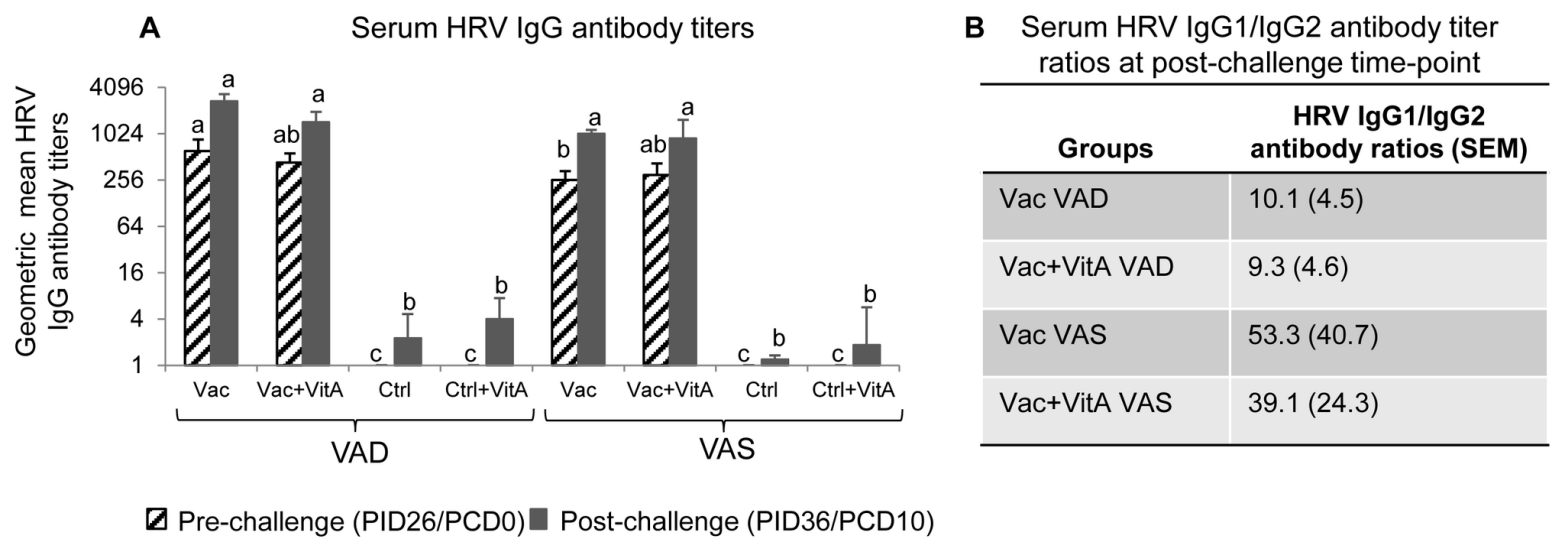
Vitamin A deficient vaccinated groups had lower HRV IgG antibody responses post-virulent HRV challenge. Geometric mean serum HRV IgG (A) titers in vitamin A deficient (VAD) and sufficient (VAS) gnotobiotic pigs vaccinated with AttHRV vaccine or placebo with or without vitamin A supplementation at pre- (PID26/PCD0) and post (PID36/PCD10)-HRV challenge time-points. Data shown as mean values ± standard error of the mean. Bars with different lower-case letters for HRV IgG antibody titers (A) at each time-point (pre- or post-challenge) differ significantly between groups (Duncans multiple range t test on log_10_ transformed data, *p* ≤ 0.05). (B) shows the HRV IgG1:IgG2 ratios for different treatment groups. Vac = 3X AttHRV vaccinated only, Vac+VitA = 3X AttHRV vaccinated + 100,000 IU of vitamin A, Ctrl = non-vaccinated and non-vitamin A supplemented, Ctrl+VitA = 3X 100,000 IU of vitamin A only. Pre-challenge: n=8-13, Post-challenge: n=4-7.

The Vac VAS group had significantly lower serum HRV IgG antibody titers compared to Vac VAD group pre-challenge (PID26/PCD0) (Figure 3A). The vaccinated VAS groups showed a similar trend for lower serum HRV IgG antibody titers post-challenge (PID36/PCD10) compared to vaccinated VAD groups (Figure 3A), which was opposite to that of serum and intestinal HRV IgA antibody responses. Further we measured HRV specific IgG1 and IgG2 antibody titers. Interestingly, higher IgG antibody titers in the vaccinated VAD groups coincided with higher (but not significantly) HRV IgG2 antibody titers in these groups compared to the vaccinated VAS groups (Figure S1). The vaccinated AS groups had 4-5 fold higher IgG1:IgG2 ratios compared to vaccinated VAD groups (Figure 3B). No consistent trends were observed for ileal and duodenal IgG ASC pre- and post-challenge suggesting that numbers of IgG ASCs at inductive and effector sites are not affected by vitamin A (Figure S2). No significant differences for total serum IgG were observed between VAD and VAS groups irrespective of vaccination, virulent HRV challenge and vitamin A supplementation (data not shown)

### Vaccinated VAS groups had significantly higher frequencies of B cells in duodenum

**Figure 4:**
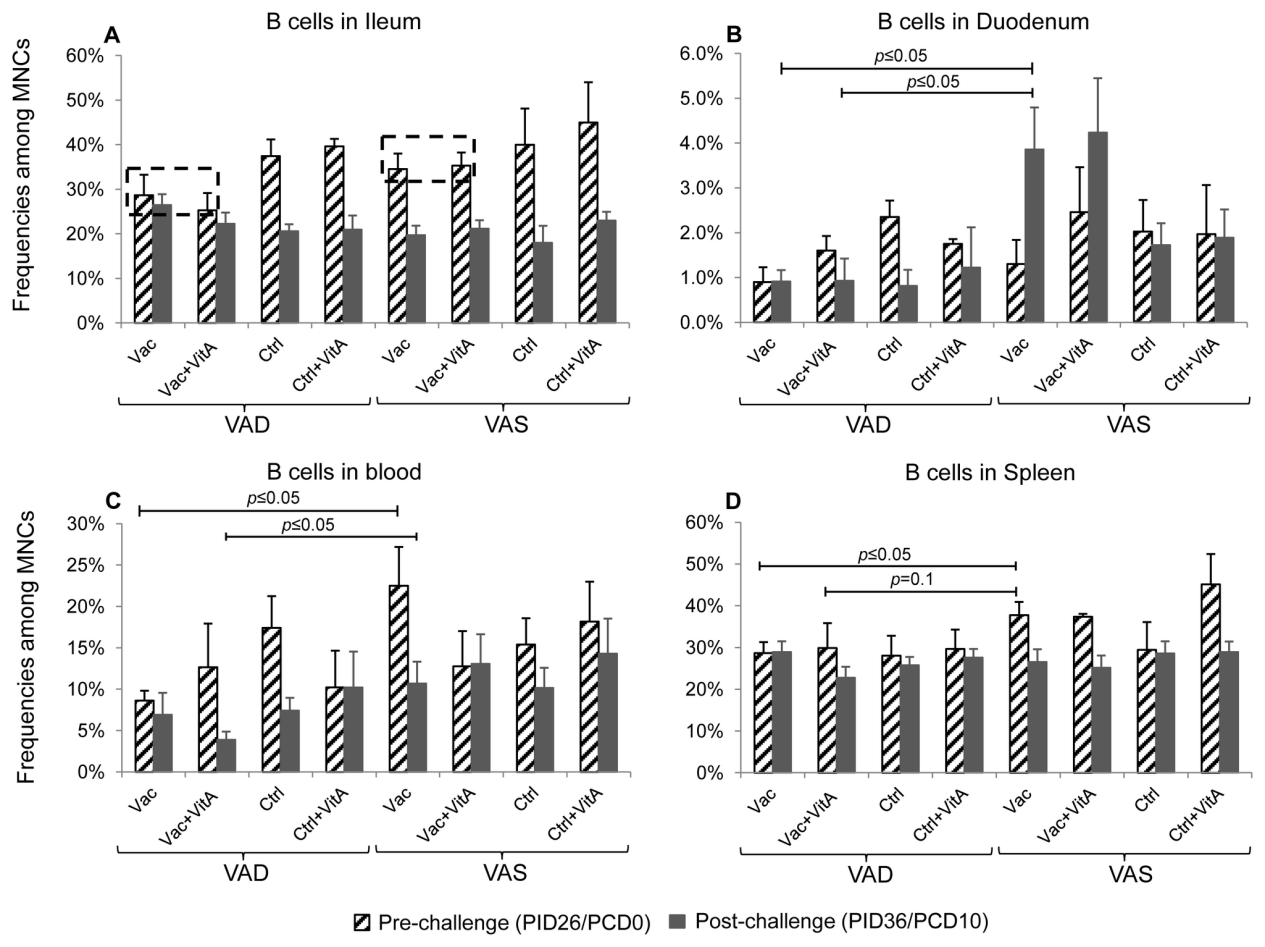
Vaccinated VAS groups had higher frequencies of B cells in ileum (pre-challenge) and duodenum (post-challenge). Mean frequency of B lymphocytes (CD21^+^CD3^-^) among mononuclear cells in vitamin A deficient (VAD) and sufficient (VAS) pigs vaccinated with AttHRV vaccine or placebo with or without vitamin A supplementation in ileum (A), duodenum (B), blood (C) and spleen (D) at pre- (PID26/PCD0) and post (PID36/PCD10)-virulent HRV challenge. Bars represent mean values and standard error of the mean. Significant differences between groups are indicated by capped lines as determined by non-parametric Kruskal-wallis rank sum test (*p* ≤ 0.05). The dotted rectangles (A) indicate trend for higher frequencies of B cells in vaccinated VAS groups compared to vaccinated VAS groups at pre-challenge. Vac = 3X AttHRV vaccinated only, Vac+VitA = 3X AttHRV vaccinated + 100,000 IU of vitamin A, Ctrl = non-vaccinated and non-vitamin A supplemented, Ctrl+VitA = 3X 100,000 IU of vitamin A only, Pre = pre-challenge (number), Post = post-challenge (number). Pre-challenge: n= 3-7, Post-challenge: n=4-7.

We investigated the frequencies of B cells (CD21^+^CD3^-^) in intestinal and systemic tissues and blood (Figure 4). Pre-challenge, a trend for higher frequencies of B cells was observed in ileum (mucosal induction site) in the vaccinated VAS groups compared to vaccinated VAD groups (Figure 4A). Post-challenge, a decrease in ileal B cells coincided with an increase in duodenal B cells in vaccinated VAS groups suggesting appropriate induction and migration of B cells to upper intestinal effector sites (Figure 4A, 4B). Interestingly, this B cell kinetics was not observed in the vaccinated VAD groups. The significant increase in duodenal B cells in the vaccinated VAS vs vaccinated VAD groups post-challenge also coincided with higher numbers of IgA ASC (at post-challenge) in the mucosal effector site (duodenum) of the former group. No effect of vitamin A supplementation was observed on intestinal B cells irrespective of vitamin A status and/or vaccination and/or virulent HRV challenge (Figure 4A, 4B).

Pre-challenge, the Vac VAD group had significantly lower frequencies of B cells in blood and spleen compared to the Vac VAS group (Figure 4C, 4D). No consistent trends were observed for distribution and frequencies of B cells in blood and spleen in different groups post-challenge irrespective of vitamin A status and/or supplementation and/or vaccination and/or challenge (Figure 4C, 4D).

### VAD vaccinated groups had lower intestinal and systemic CD4 Treg cells post-challenge

**Figure 5:**
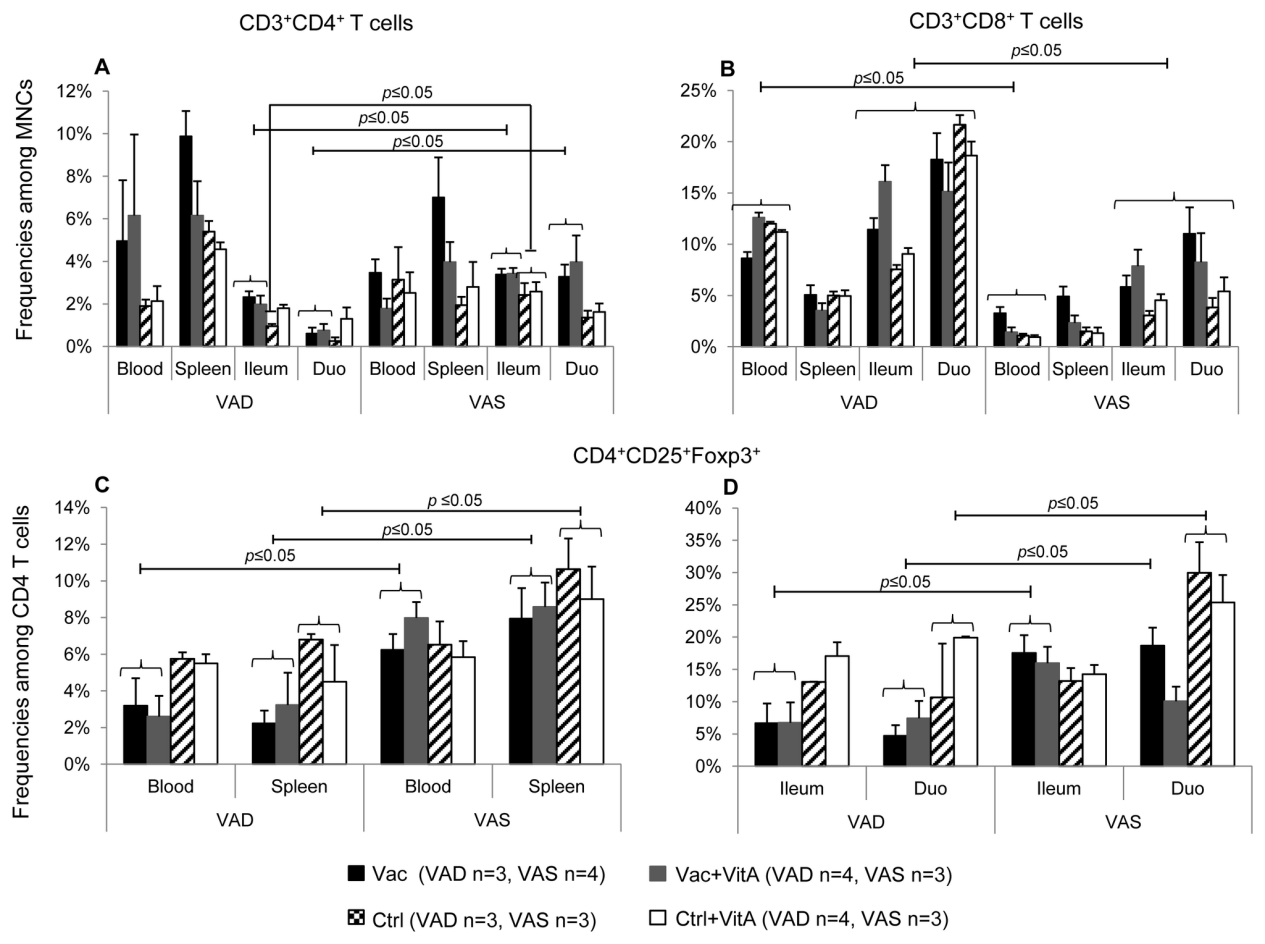
Vitamin A sufficient vaccinated groups had higher intestinal and systemic T regulatory cells post-virulent HRV challenge. Mean frequencies CD3^+^CD4^+^ (A) and CD3^+^CD8^+^ (B) T cells among mononuclear cells (MNCs) and CD4^+^CD25^+^Foxp3^+^ (C,D) among CD4 T cells in vitamin A deficient (VAD) and sufficient (VAS) pigs vaccinated with AttHRV vaccine or placebo with or without vitamin A supplementation in blood, spleen, ileum and duodenum post-virulent HRV challenge Bars represent mean values and standard error of the mean. Significant differences between groups are indicated by capped lines as determined by non-parametric Kruskal-wallis rank sum test (*p* ≤ 0.05). Vac = 3X AttHRV vaccinated only, Vac+VitA = 3X AttHRV vaccinated + 100,000 IU of vitamin A, Ctrl = non-vaccinated and non-vitamin A supplemented, Ctrl+VitA = 3X 100,000 IU of vitamin A only.

T cell responses were determined by flow cytometry in 2 of the 4 sets of piglets derived from VAD and VAS sows (Figure 5). Post-challenge, vaccinated VAS groups had significantly higher frequencies of T helper cells (CD3^+^CD4^+^) in ileum and duodenum compared to the vaccinated VAD groups (Figure 5A) suggesting that normal vitamin A levels are necessary for generation of appropriate T cell responses and subsequent antibody responses to oral AttHRV vaccine and virulent HRV challenge. Non-vaccinated VAS groups had significantly higher frequencies of ileal T helper cells compared to the Ctrl VAD group post-challenge (Figure 5A). In contrast to T helper cells, VAD groups had significantly higher frequencies of cytotoxic T cells (CD3^+^CD8^+^) in intestinal tissues compared to VAS groups post-challenge (Figure 5B), irrespective of vaccination, suggesting cell type-specific effects of vitamin A on T cell subsets. Moreover, higher frequencies of CD3^+^CD8^+^ T cells were also observed in blood of VAD groups compared to VAS groups (Figure 5B).

Retinoic acid plays an important role in induction of Treg cells [12]. Post-challenge, vaccinated VAS groups had significantly higher frequencies of CD4^+^CD25^+^Foxp3^+^ T cells in blood and spleen compared to vaccinated VAD groups, irrespective of vitamin A supplementation (Figure 5C). Similarly, in ileum CD4 Treg cells were significantly higher in the vaccinated VAS groups compared to the vaccinated VAD groups post-challenge (Figure 5D) suggesting that intestinal and systemic regulatory responses were compromised in VAD conditions. In duodenum, no significant differences were observed between the Vac+VitA VAS group and the Vac and Vac+VitA VAD groups for CD4 Treg cells post-challenge (Figure 5D). However, the Vac VAS group had significantly higher frequencies of CD4 Treg cells compared to the vaccinated VAD groups (Figure 5D). In spleen and duodenum, VAD control groups had lower frequencies of CD4 T reg cells compared to the VAS control groups, irrespective of vitamin A supplementation (Figure 5C, 5D).

### Vitamin A deficiency induced pro-inflammatory immune responses

**Figure 6:**
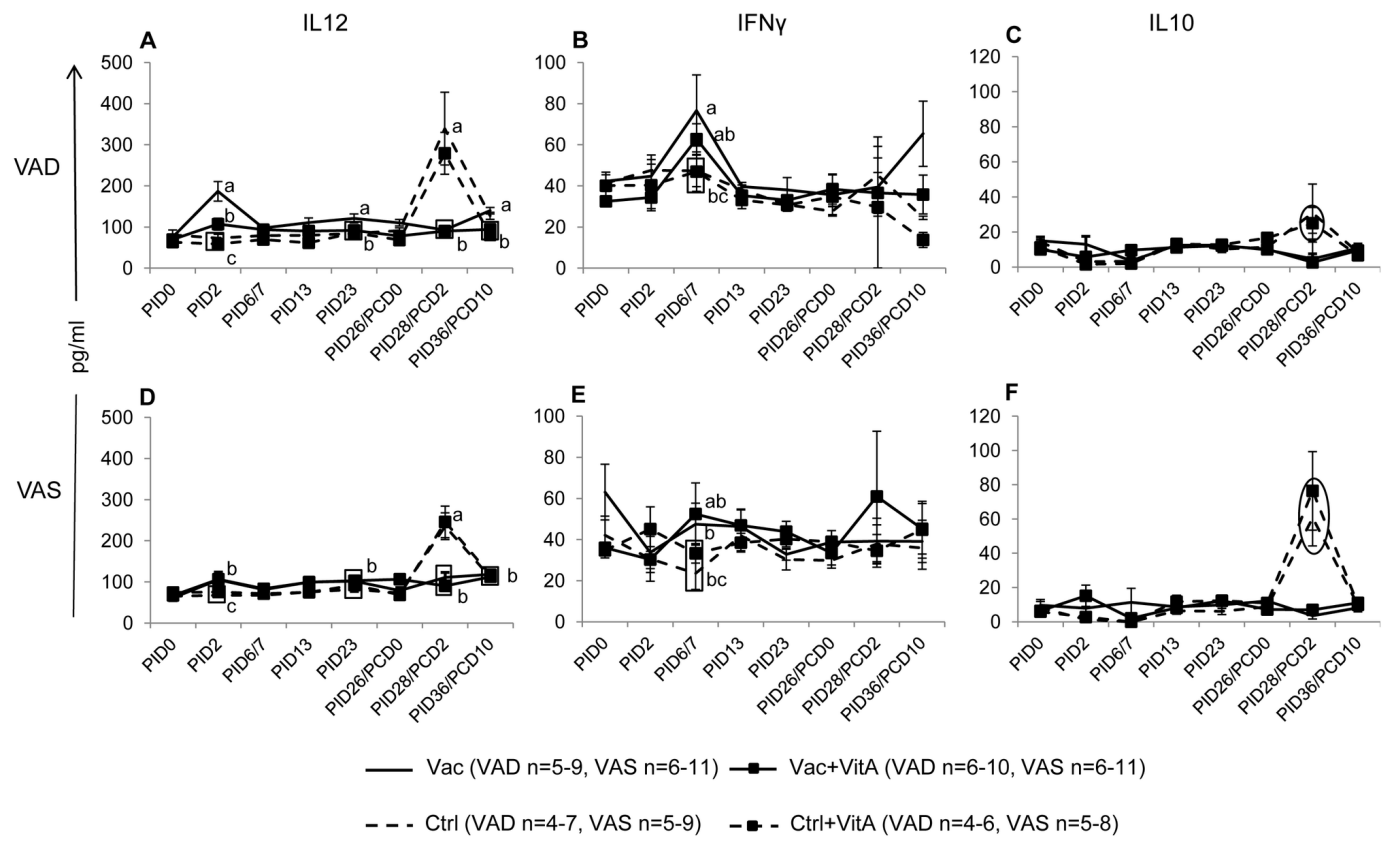
Vitamin A deficiency induced pro-inflammatory immune responses. Mean concentrations (± SEM) of IL12 (A,D; Th1), IFNγ (B,E; Th1) and IL10 (C,F; anti-inflammatory) cytokines in serum of vitamin A deficient (VAD) and sufficient (VAS) pigs vaccinated with AttHRV vaccine or placebo with or without vitamin A supplementation. Data shown as mean values ± standard error of the mean. Different lower-case letters indicate statistically significant difference (non-parametric Kruskal-wallis rank sum test*, p* ≤ 0.05) among the treatment groups belonging to VAD and VAS pigs at the same time-point for each cytokine. The circles (C,F) indicate 2-3 fold higher IL10 in VAS control groups compared to VAD control groups. Vac = 3X AttHRV vaccinated only, Vac+VitA = 3X AttHRV vaccinated + 100,000 IU of vitamin A, Ctrl = non-vaccinated and non-vitamin A supplemented, Ctrl+VitA = 3X 100,000 IU of vitamin A only.

Pro- and anti-inflammatory responses associated with vitamin A status, AttHRV vaccination and virulent HRV challenge were assessed by determining serum IFNγ, IL12 and IL10 levels at multiple time points pre- and post-challenge (Figure 6). Mean serum IL12 concentrations showed an interesting trend and coincided inversely to hepatic vitamin A levels in the different groups (Figure 6A, 6D). The Vac VAD group had significantly higher serum IL12 levels compared to the Vac+VitA VAD group and vaccinated VAS groups (Figure 6A, 6D). This finding suggests that vitamin A deficiency results in altered cytokine responses to AttHRV vaccine. Serum IFNγ exhibited a similar trend to that of IL12 pre-challenge. The Vac VAD group had significantly higher serum IFNγ at PID6 compared to the Vac VAS group (Figure 6B, 6E). However, no significant differences were observed post-challenge in the different groups irrespective of vitamin A status. The IL10 (anti-inflammatory) was 2-3 fold higher in the VAS control groups (Ctrl: 61pg/ml and Ctrl+VitA:76 pg/ml) compared to the VAD control groups (Ctrl:30 pg/ml and Ctrl+VitA: 24pg/ml) post-challenge (Figure 6C, 6F) suggesting an anti-inflammatory microenvironment induced by adequate vitamin A levels post-virulent HRV challenge.

### VAD control groups had higher serum IFNα post-virulent HRV challenge

**Figure 7:**
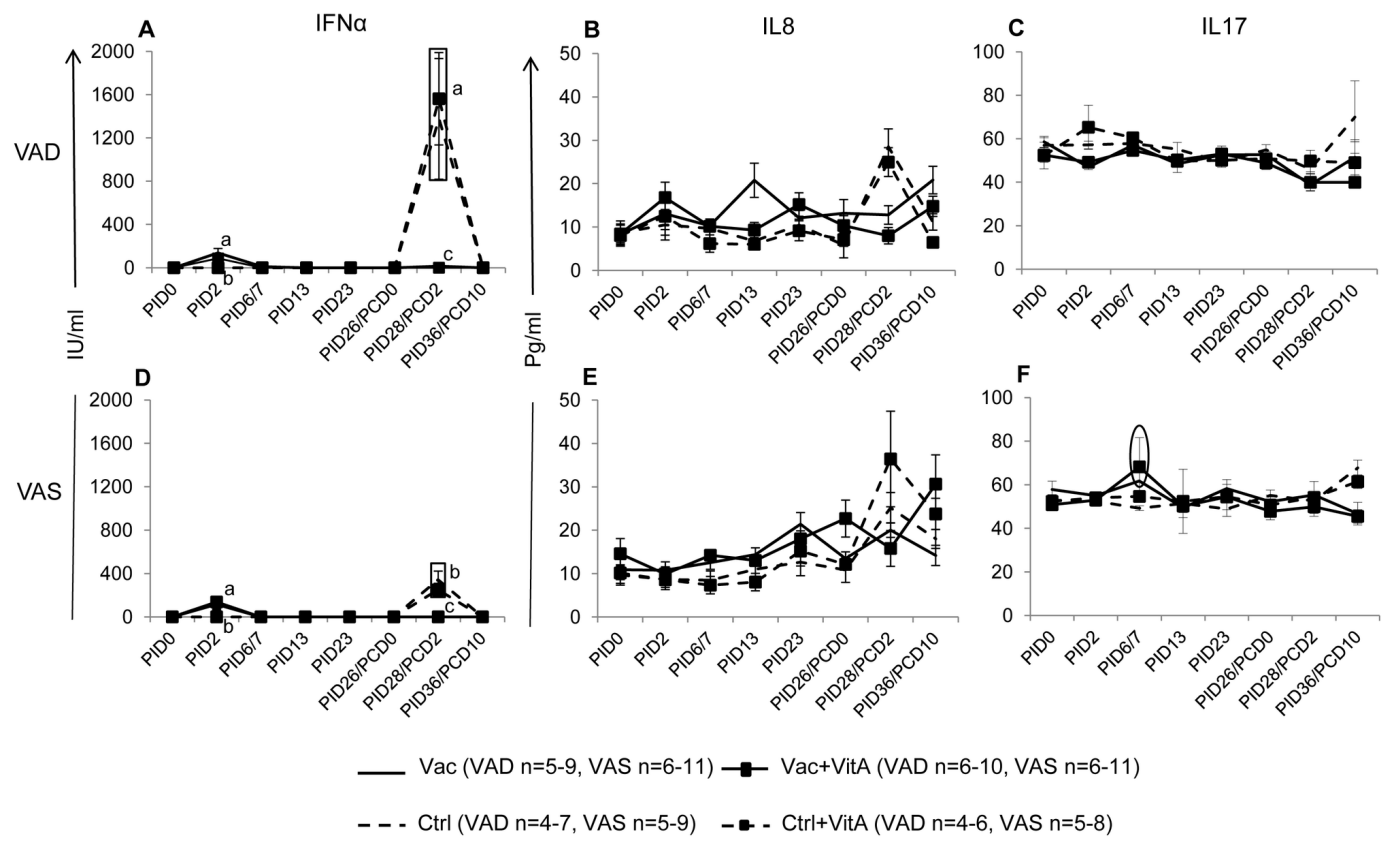
Summary of innate and Th17 cytokine responses. Mean concentrations (± SEM) of IFNα (A,D; innate), IL8 (B,E; innate) and IL17 (C,F; Th17) cytokines and chemokines in serum of vitamin A deficient (VAD) and sufficient (VAS) pigs vaccinated with AttHRV vaccine or placebo with or without vitamin A supplementation. Data shown as mean values ± standard error of the mean. Different lower-case letters indicate statistically significant difference (non-parametric Kruskal-wallis rank sum test*, p* ≤ 0.05) among the treatment groups belonging to VAD and VAS pigs at the same time-point for each cytokine. The circle (f) indicates increase in IL17 in vaccinated VAS groups compared to VAS control groups. Vac = 3X AttHRV vaccinated only, Vac+VitA = 3X AttHRV vaccinated + 100,000 IU of vitamin A, Ctrl = non-vaccinated and non-vitamin A supplemented, Ctrl+VitA = 3X 100,000 IU of vitamin A only.

At PID2, no significant differences were observed for serum IFNα between different groups irrespective of vitamin A status or vitamin A supplementation between vaccinated groups (Figure 7A, 7D). Post-challenge, VAD control pigs, irrespective of vitamin A supplementation, had significantly higher serum IFNα compared to the counterpart VAS control pigs, which coincided with significantly higher fecal HRV shedding and diarrhea severity in the VAD control pigs post-challenge (Figures 7A,7D,1A,1B, Table 1). Serum IFNα levels detected by ELISA coincided with IFNα concentrations determined by biological assay [33]. No consistent trends were observed for IL8 pre- or post-challenge except at PID13 (after second AttHRV vaccination), when significantly higher IL8 was observed in the Vac VAD group compared to the other vaccinated VAD and VAS groups (Figure 7B, 7E). Serum IL17 showed an increase in vaccinated VAS groups at PID6 compared to the control VAS groups irrespective of vitamin A supplementation (Figure 7F). This increase in IL17 was not observed in the vaccinated VAD groups compared to the control VAD groups irrespective of vitamin A supplementation (Figure 7C). 

### Continued supplementation of vitamin A in a pilot study showed some beneficial effects in AttHRV vaccinated pigs post-virulent HRV challenge

**Figure 8:**
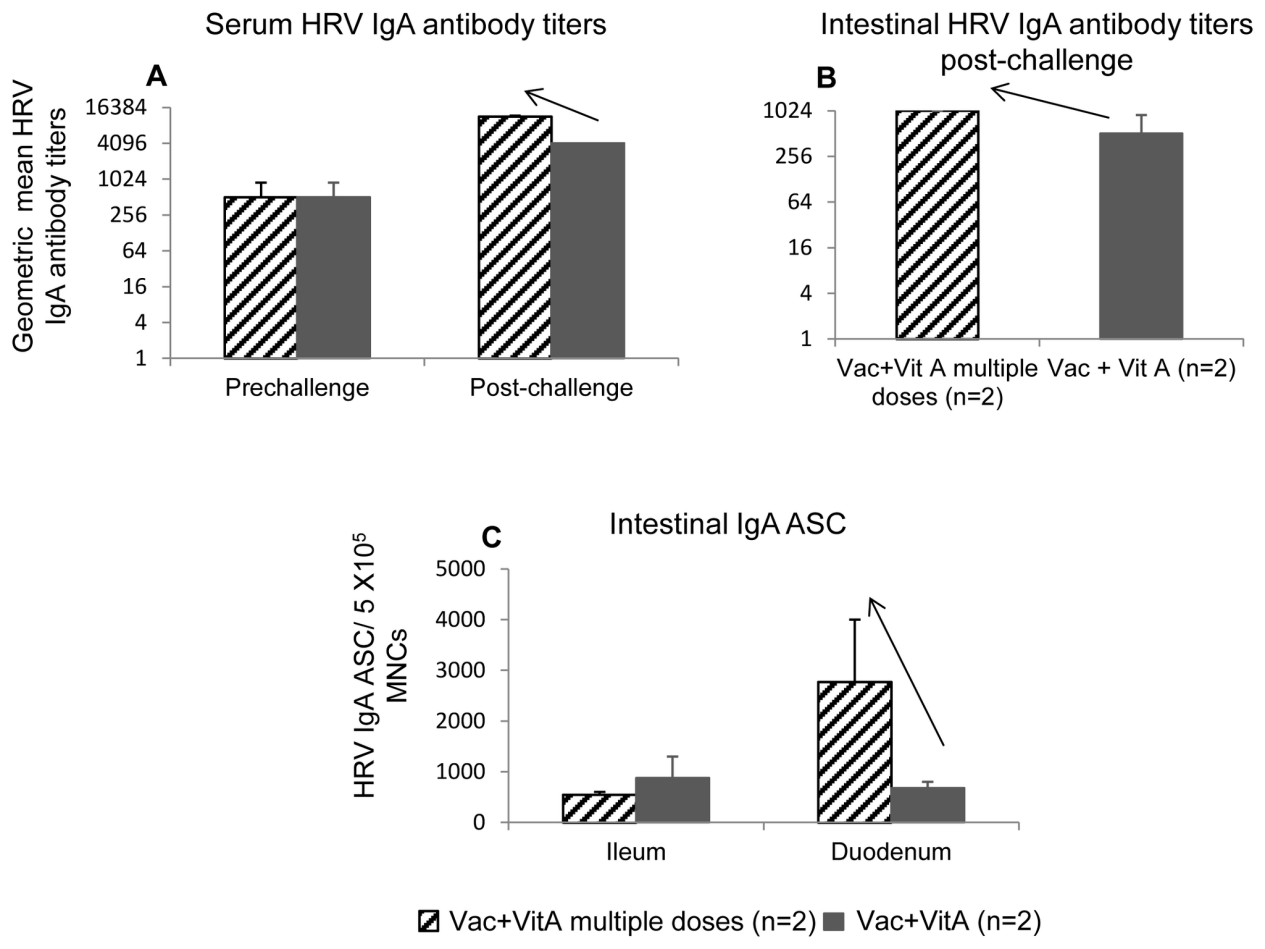
Summary of pilot data for different regimens for vitamin A supplementation. Geometeric mean titers of serum (A) and intestinal (B) HRV IgA antibody titers and mean intestinal HRV IgA antibody secreting cells (C) in AttHRV vaccinated pigs with supplemented of 50,000 IU for 11 days (Vac+VitA multiple doses, n=2) or 100,000 IU at each vaccine time-point (Vac+VitA, n=2). Data shown as mean values ± standard error of the mean. Arrows in a, b, c and d indicate increase in these parameters for Vac+VitA multiple dose compared to Vac+VitA group. Vac+VitA = 3X AttHRV vaccinated + supplemental vitamin A. No statistical analysis was done because of low numbers of pigs tested per treatment in this pilot study.

Lack of consistent immunomodulatory/compensatory effects of vitamin A supplementation in VAD piglets observed in our study can be due to multiple reasons, including non-optimal regimen or doses of supplementation. Therefore, in a pilot trial we compared WHO recommended vitamin A supplementation schedule (used in our study, n=2) with more frequent and lower dose of supplemental vitamin A (50,000 IU/each time point, n=2). In this new regimen, we supplemented pigs born to normal sows for 11 days continuously starting 2 days before first vaccine dose and ending at the second vaccine dose. Pigs that received continued supplemental vitamin A had higher serum and intestinal IgA HRV antibody titers post-challenge compared to those that received vitamin A at immunization contact (Figure 8A, 8B). Moreover this also coincided with higher duodenal IgA ASC (Figure 8C) post-challenge in the pigs that received continued vitamin A compared to those that received only 3 doses.

## Discussion

Vitamin A deficiency in children is a significant health problem in impoverished countries and prophylactic supplemental vitamin A recommended by WHO has reduced the incidence of diarrhea associated mortality and morbidity in these countries [7,38]. To investigate interactions between vitamin A status, supplemental vitamin A, AttHRV vaccine and virulent HRV infection, we established a VAD gnotobiotic pig model [33]. In this study in a relevant animal model for HRV challenge studies, we show that vitamin A deficiency not only affects severity of HRV induced diarrhea, but it also impairs both mucosal and systemic adaptive B and T lymphocyte responses to an AttHRV vaccine, which is genotypically similar to commercially available monovalent HRV vaccine, Rotarix (G1P[8]).

We generated subclinical VAD pigs by controlling dietary vitamin A intake of sows (equivalent to pregnant women with low vitamin A) and created a scenario similar to that of children from developing countries [5]. Subclinical vitamin A deficiency in the control VAD pigs (lowest hepatic vitamin A levels among all groups) coincided with severe diarrhea and higher fecal HRV shedding post-challenge compared to the control VAS pigs, suggesting that improving vitamin A status in VAD children may reduce the severity of HRV infection and associated mortality and costs of hospitalization. Similar to our results, adult VAD CD-1 mice showed higher RV excretion in the stool and higher intestinal pathological lesions compared to the control group, post-challenge [39]. Unexpectedly in our study, 3 dose vitamin A supplementation in bolus form was not helpful in improving the clinical situation of VAD pigs. Similarly, a recent study showed no effect of supplemental vitamin A (50,000 IU given with BCG vaccine) in infants on rotavirus associated diarrhea [40]. In contrast, studies conducted in Mexico have shown beneficial effects of supplemental vitamin A in older children against norovirus GII and enteropathogenic *E. coli* diarrhea [41,42] suggesting that effects may vary with initial vitamin A status, age, infectious agents and geographical locations.

Higher severity of RV diarrhea in the VAD control group coincided with significantly higher serum IFNα (innate), slightly increased IL12 (Th1, only Ctrl group), and lower IL10 (anti-inflammatory) levels compared to VAS control groups post-virulent HRV challenge suggesting higher stimulation of innate and adaptive pro-inflammatory cytokine responses to HRV infection in VAD groups. Moreover, subclinical vitamin A deficiency in control pigs resulted in imbalanced distribution of innate and adaptive cells. VAD control groups had higher frequencies of CD8 T cells in all the tissues examined and lower frequencies of CD4 T cells (Ctrl group only) in the ileum post-challenge, and higher frequencies of plasmacytoid DCs in intestinal tissues and conventional DCs in all the tissues examined pre-challenge, compared to VAS control pigs [33], suggesting dysregulation of overall immune responses under reduced vitamin A conditions. Similar to our study, lower frequencies of CD4 T cells and increased frequencies of CD8 T cells in intestinal tissues and spleen have been reported in VAD mice and rat models [43,44]. Aberrant frequencies and distribution of innate and adaptive cells and lower Treg cells in VAD pigs may affect both humoral and cell-mediated immune responses to virulent HRV challenge. The higher diarrhea severity and shedding in control VAD groups can also be attributed partly to the fundamental role of vitamin A in maintaining the integrity of gastrointestinal epithelium [9,45]. Clinical trials in children have suggested that supplementation of vitamin A in children with diarrhea results in improved intestinal integrity as determined by dual sugar permeability tests [9,46].

Interestingly in our study, IFNα, which is an innate anti-viral cytokine, was not associated with protection but showed strong association with HRV replication as indicated by higher fecal shedding titers post-challenge in VAD control groups. Our findings are consistent with the observation in children, where vomition episodes and rotavirus diarrhea significantly correlated with IFNα levels [47]. Timing of IFNα induction may determine the quality of its anti-RV activity and is critical in understanding its effects. For example, IFNα administered before RV infection reduced RV diarrhea in pigs and calves [48,49]. However, pretreatment with IFNα in neonatal mice had no effect on virus shedding, suggesting species specific effects [50]. Furthermore, recent studies have questioned the role of IFNα in RV clearance in a mouse model and have suggested that these effects may vary between homologus and heterologous RV infections [51].

No significant differences were observed in the protection rates against diarrhea between VAD and VAS vaccinated groups. However, diarrhea severity scores and shedding rates were higher in the Vac VAD group compared to the vaccinated VAS groups. This coincided with lower serum HRV IgA antibody titers and duodenal IgA ASC post-challenge in the vaccinated VAD groups compared to the vaccinated VAS groups, suggesting that subclinical vitamin A deficiency may affect generation of anamnestic immune responses to virulent HRV infection in vaccinated children and result in reduced efficacy of current RV vaccines. Serum and intestinal/fecal HRV IgA antibody titers are correlates of protection in children in some studies and in gnotobiotic pigs [25,35,52]. Increased duodenal IgA ASC post-challenge in VAS vaccinated groups also coincided with higher frequencies of B cells in duodenum post-challenge suggesting effective homing of activated B cells to gut mucosa. We could not specifically investigate expression of intestinal homing receptor α4β7 on the effector B cells due to lack of porcine specific or cross-reactive antibodies. Specialized CD103^+^ (αEβ7) intestinal DCs, which express retinal dehydrogenase enzymes (RALDH), can metabolize vitamin A and synthesize RA. This RA is important to imprint gut homing of effector B cells by promoting expression of α4β7 on effector B cells [10,11]. Previously, we have shown that the frequencies of the intestinal CD103^+^ expressing DCs were lower in the VAD groups compared to VAS groups both pre and post-challenge [33], which may affect homing of HRV IgA ASC to intestinal tissues and the magnitude of antibody responses. Moreover, lower frequencies of intestinal T helper cells in VAD vaccinated groups associated with aberrant innate immune responses may affect antigen presentation and subsequent induction of B cell responses to AttHRV vaccine and virulent HRV challenge, further contributing to lower vaccine efficacy. Similar to the trends for total T helper cells in intestinal tissues, we also observed lower frequencies of Treg cells in the intestinal tissues of vaccinated VAD groups compared to the vaccinated VAS groups. These findings suggest that Treg cell numbers depend on vitamin A status of the host and that vitamin A deficiency adversely affected immunoregulatory responses. Lower frequencies of Treg cells in vaccinated VAD animals may have contributed to the higher inflammatory immune responses and exacerbated RV induced pathology and resulted in higher diarrhea severity scores and shedding rates. Similar to our result, *in vitro* and *in vivo* studies have shown induction of Treg cells by RA [12,53,54]. Thus, subclinical (or marginal) vitamin A deficiency in children may result in lower B and T cell responses to current oral RV vaccines and may contribute to the lower vaccine efficacy in developing countries.

The HRV IgA and IgG antibody titers in the vaccinated VAD and VAS groups relied on vitamin A status, post-challenge. The Vac+VitA VAS group had the highest mean serum and intestinal HRV IgA antibody titers and lowest mean serum HRV IgG antibody titers of all vaccinated groups post-challenge and vice-versa for the Vac VAD group suggesting that vitamin A deficiency may have affected the balance of HRV IgA/IgG isotypes. These findings are in agreement with the previous studies conducted in VAD animal models. Severe and mild influenza virus challenge in adult VAD rats resulted in higher virus-specific serum IgG antibodies and lower virus-specific salivary IgA antibodies [17]. Furthermore, VAD rats supplemented with higher amounts of vitamin A had higher influenza-specific salivary IgA antibodies and lower serum IgG antibodies compared to the VAD group on a control diet [55]. Similarly, adult VAD mice inoculated intranasally with Sendai virus had lower virus-specific nasal IgA and higher virus-specific bronchial IgG antibodies compared to control mice [18,19]. Thus, the effect of vitamin A on the balance of IgA and IgG antibody isotypes appears to be maintained across species and diverse pathogens.

In our study, we observed higher concentrations of pro-inflammatory cytokines (IFNγ and IL12) post-AttHRV inoculation in the Vac VAD group compared to the vaccinated VAS groups, suggesting a Th1 induced microenvironment in VAD pigs, which may have contributed to higher diarrhea severity. No differences were observed in anti-inflammatory serum IL10 levels between different vaccinated groups post-AttHRV inoculation, but post-virulent HRV challenge, higher concentrations were observed in the VAS compared to VAD control groups suggesting more regulatory and less inflammatory microenvironment in the presence of adequate levels of vitamin A. Similar to our results, a clinical study conducted in Venezuelan children showed lower levels of serum IL10 associated with VAD disorders [56]. Moreover, previous studies in VAD animal models (rat and mice) and vitamin A deficiency in children have shown a predisposition to type 1 (Th1) immune responses suggesting a bias for pro-inflammatory responses in VAD conditions, similar to our results [55,57,58].

We successfully established a VAD pig model but could not comprehensively show that supplementation of vitamin A (with the schedule and regimen based on recommendations by WHO for measles and DPT vaccines) could help to restore the dysregulated immune responses to AttHRV vaccine and/or virulent HRV challenge. We could demonstrate only limited adjuvant effects of this regimen on AttHRV vaccine in VAD conditions, as indicated by increased duodenal IgA ASC (3-fold) at pre-challenge and significantly decreased serum IL12 levels at PID2 in the Vac+VitA VAD group compared to the non-supplemented vaccinated VAD group. Therefore, we compared WHO recommended vitamin A dosage schedule with a different schedule for vitamin A supplementation (continued 50,000 IU of supplemental vitamin A for 12 days) in a pilot study in normal (~VAS) pigs. Our preliminary studies showed that this prolonged dosing schedule resulted in higher immune responses. These findings suggest that changing the supplementation regimen and doses for vitamin A may result in more beneficial effects on immune responses to RV vaccines and infection in children. Lower responses to supplemental vitamin A in VAD animals may also be attributed to lower expression of RARα or RALDH enzymes by immune cells which may result in inherent limitations for metabolizing vitamin A and its subsequent beneficial effects [33,59], irrespective of vitamin A supplementation regimen and dosage.

In conclusion, our data demonstrate that vitamin A deficiency results in more severe HRV diarrhea. We showed that vitamin A status determines adaptive B and T cell responses to AttHRV vaccine and virulent HRV challenge. Higher vitamin A levels were associated with higher serum HRV IgA antibody titers, intestinal HRV IgA ASC responses and Treg cells, and lower diarrhea severity scores in vaccinated VAS groups, highlighting a critical link of micronutrient status with RV vaccine protective efficacy. Furthermore, lower vitamin A levels were associated with higher systemic inflammatory responses. These findings suggest that prevalent vitamin A deficiency in children from developing countries is an important factor influencing efficacy of oral HRV vaccines. However, supplementation of high dose vitamin A by the oral route at immunization contact did not result in optimum adjuvant effects of vitamin A or restoration of immune responses to virulent HRV infection in VAD conditions. Because of the observed differences between VAD and VAS pigs, vitamin A supplementation may be useful in children in developing countries. However, a different regimen and doses of supplemental vitamin A should be tested further to enhance HRV vaccine efficacy and to reduce the associated HRV morbidity and mortality.

## Supporting Information

Figure S1 **HRV specific IgG1 and IgG2 antibody responses in VAD and VAS vaccinated groups.** Geometric mean serum HRV IgG1 (A) and IgG2 (B) titers in vitamin A deficient (VAD) and sufficient (VAS) gnotobiotic pigs vaccinated with AttHRV vaccine or placebo with or without vitamin A supplementation at pre- (PID26/PCD0) and post (PID36/PCD10)-HRV challenge time-points. Data shown as mean values ± standard error of the mean. The arrow (in B) indicates lower mean HRV IgG antibody titers in vaccinated VAS groups compared to vaccinated VAD groups at PID36/PCD10 (post-challenge). Vac = 3X AttHRV vaccinated only, Vac+VitA = 3X AttHRV vaccinated + 100,000 IU of vitamin A. Pre-challenge: n=8-13, Post-challenge: n=4-7.
(TIF)

Figure S2 **HRV specific IgG antibody and antibody secreting cell responses.** Mean intestinal HRV IgG antibody secreting cells in vitamin A deficient (VAD) and sufficient (VAS) gnotobiotic pigs vaccinated with AttHRV vaccine or placebo with or without vitamin A supplementation at pre- (PID26/PCD0) (a) and post (PID36/PCD10)-HRV challenge (b) time-points. Data shown as mean values ± standard error of the mean. Significant differences between groups for HRV IgG ASC are indicated by capped lines as determined by non-parametric Kruskal-wallis rank sum test (*p* ≤ 0.05). Vac = 3X AttHRV vaccinated only, Vac+VitA = 3X AttHRV vaccinated + 100,000 IU of vitamin A, Ctrl = non-vaccinated and non-vitamin A supplemented, Ctrl+VitA = 3X 100,000 IU of vitamin A only. Pre-challenge: n= 3-7, Post-challenge: n=4-7.
(TIF)
