# Supplementary figures and images for: Vitamin A Deficiency Impairs Adaptive B and T Cell Responses to a Prototype Monovalent Attenuated Human Rotavirus Vaccine and Virulent Human Rotavirus Challenge in a Gnotobiotic Piglet Model
Source: PLoS One. 2013 Dec 2;8(12):e82966. doi: 10.1371/journal.pone.0082966 (PMC3846786; doi:10.1371/journal.pone.0082966)

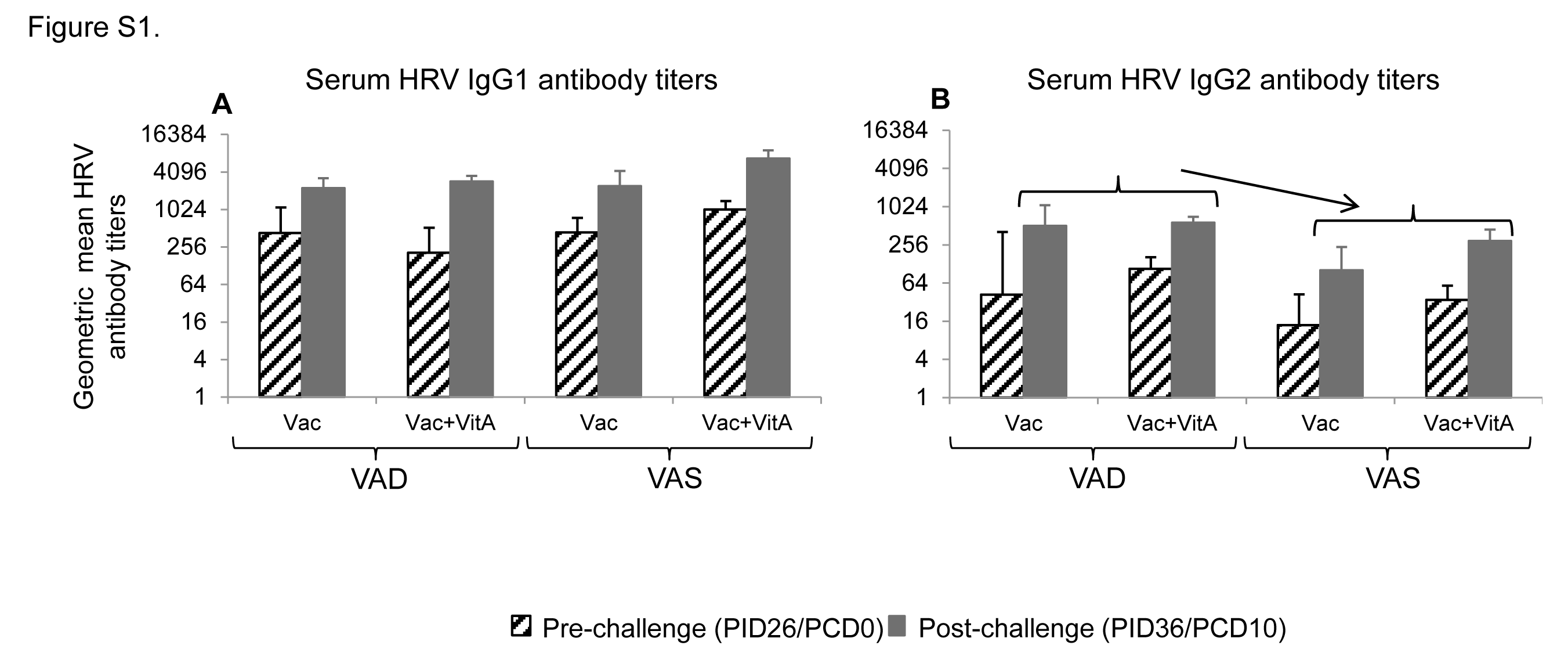

Supplement: Figure S1 — HRV specific IgG1 and IgG2 antibody responses in VAD and VAS vaccinated groups. Geometric mean serum HRV IgG1 (A) and IgG2 (B) titers in vitamin A deficient (VAD) and sufficient (VAS) gnotobiotic pigs vaccinated with AttHRV vaccine or placebo with or without vitamin A supplementation at pre- (PID26/PCD0) and post (PID36/PCD10)-HRV challenge time-points. Data shown as mean values ± standard error of the mean. The arrow (in B) indicates lower mean HRV IgG antibody titers in vaccinated VAS groups compared to vaccinated VAD groups at PID36/PCD10 (post-challenge). Vac = 3X AttHRV vaccinated only, Vac+VitA = 3X AttHRV vaccinated + 100,000 IU of vitamin A. Pre-challenge: n=8-13, Post-challenge: n=4-7. (TIF) [file pone.0082966.s001.tif]

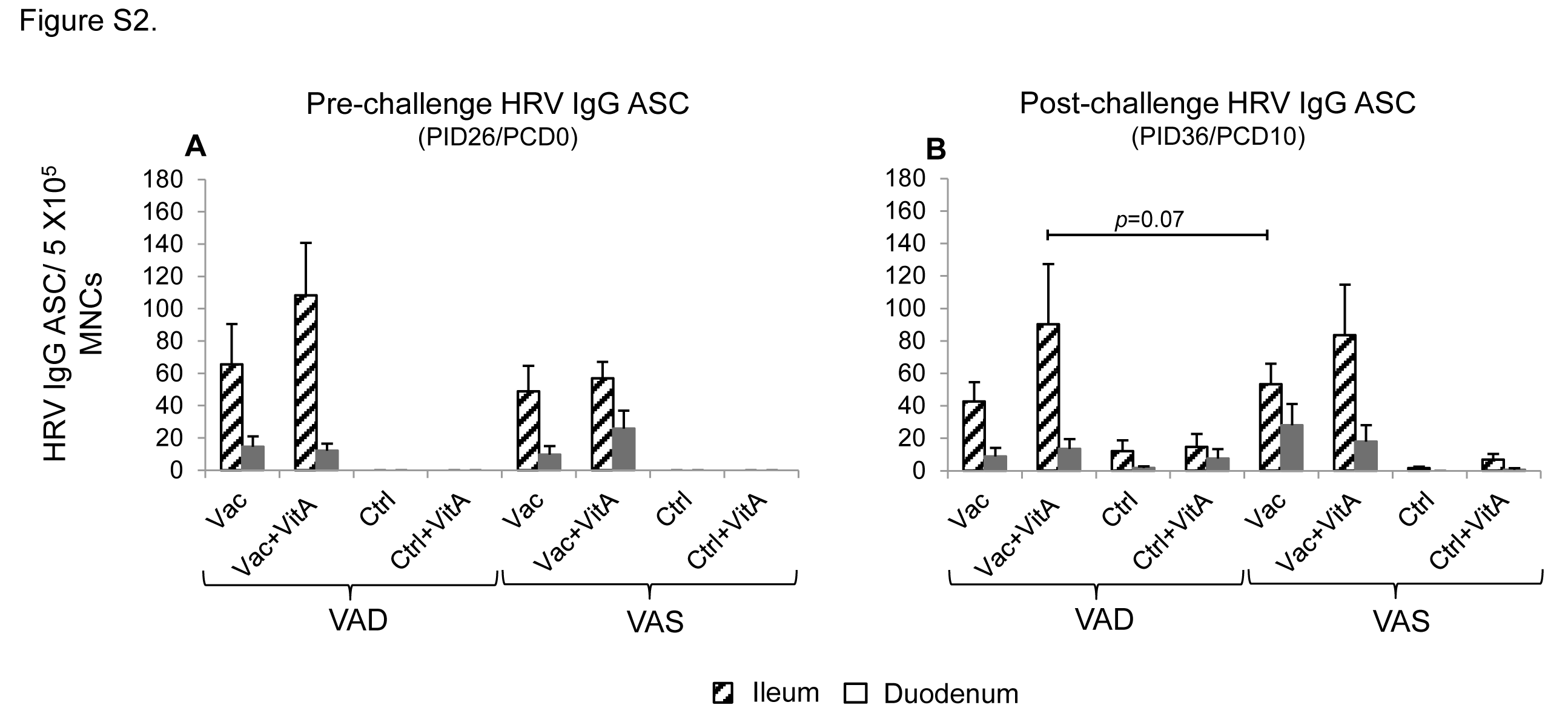

Supplement: Figure S2 — HRV specific IgG antibody and antibody secreting cell responses. Mean intestinal HRV IgG antibody secreting cells in vitamin A deficient (VAD) and sufficient (VAS) gnotobiotic pigs vaccinated with AttHRV vaccine or placebo with or without vitamin A supplementation at pre- (PID26/PCD0) (a) and post (PID36/PCD10)-HRV challenge (b) time-points. Data shown as mean values ± standard error of the mean. Significant differences between groups for HRV IgG ASC are indicated by capped lines as determined by non-parametric Kruskal-wallis rank sum test (p ≤ 0.05). Vac = 3X AttHRV vaccinated only, Vac+VitA = 3X AttHRV vaccinated + 100,000 IU of vitamin A, Ctrl = non-vaccinated and non-vitamin A supplemented, Ctrl+VitA = 3X 100,000 IU of vitamin A only. Pre-challenge: n= 3-7, Post-challenge: n=4-7. (TIF) [file pone.0082966.s002.tif]
